# The future of *PLOS Biology*

**DOI:** 10.1371/journal.pbio.3000707

**Published:** 2020-03-31

**Authors:** Nonia Pariente, on behalf of the PLOS Biology Staff Editors

**Affiliations:** Public Library of Science, San Francisco, California, United States of America and Cambridge, United Kingdom

Established over 15 years ago, *PLOS Biology* pioneered open access publishing in the life sciences at a time when most of the literature remained locked behind paywalls. As we look to the future, we reinvigorate our mission to act as a catalyst for open science and accelerate change in scientific communication for the benefit of science and scientists.

*PLOS Biology*, as the flagship PLOS journal in the life sciences, is a selective journal that aims to give voice to significant advances that will be widely read, built upon and drive future discovery. With the increasing volume of scientific literature and increasing time demands on researchers, the role of selective journals as a means to access the most important advances remains crucial. What constitutes a significant advance, however, is a matter of debate. At *PLOS Biology*, we consider that significant advances are those that push science forward in a sizeable, meaningful way, also when they report, for example, exciting but preliminary findings, null or negative results. Scientific discovery begins with interesting research questions and scientific publication should reflect this process. We will thus shift the focus of our initial editorial assessment from the perceived importance of the final results, to increase the emphasis on the research question being asked, the approach undertaken and the quality of execution, regardless of outcome. This will help give visibility to null or negative results, which can provide as much advance as studies more traditionally considered for publication. Not all negative or null results are equally insightful or provide the same degree of conceptual advance, and our criteria for publication will continue to be selective. However, these types of studies are often undervalued in the publication process and with this redefinition of our selectivity criteria we hope to redress this bias.

Publication should be an integral part of the research process, rather than something that interrupts and may even be seen to hinder discovery. In addition to our more traditional article types, we will provide researchers with options to publish their research as it unfolds by introducing Preregistered Research Articles (PRAs) and linked Discovery Reports and Update Articles. PRAs, also known as registered reports, undergo peer review immediately after the study design stage, before experiments are conducted. If a study is invited, results and discussion are subsequently added to create a single, fully integrated article. Peer review then focuses on adherence to the approved approach, the appropriateness of any deviations, and the accuracy of the conclusions, but not on the nature of the results obtained. This approach, especially useful for hypothesis-driven research, prevents publication bias and helps ensure the best possible study design at a time when modifications are still possible. Linked Discovery Reports and Update Articles give researchers the flexibility of publishing their initial findings as a first step, thereby helping the field move forward earlier, providing credit earlier, and alleviating the “full story tyranny” or pressure of having to wait to fully develop a story before publication. Discovery Reports describe novel and intriguing initial findings, confirmed by independent methods, that have the potential to lead to a significant new result for the field. As the community builds on this finding, the original authors or other research groups can augment the initial report with linked Update Articles that add mechanistic understanding, physiological relevance or other new insights. Several Update Articles can be linked to the same Discovery Report, allowing for a series of interconnected findings and resulting in scientific communication that more closely mirrors the research process.

Since our launch in 2003, we have built strong connections with many communities, and we will now focus on expanding our reach to areas that are less well represented at the journal. As an open and inclusive journal, we aim to provide a home for exciting advances in all areas of the life sciences. No field is out of our scope or interest and we will actively work towards extending our reach, including by expanding our Editorial Board and advocacy in less represented areas. If you have work that significantly advances its field or is at the crossroads between biology and related fields, regardless of the biological discipline, we would love to hear from you.

A defining characteristic of PLOS, and indeed of *PLOS Biology*, is our commitment to Open Science. We will continue to work with the communities we serve towards furthering our initiatives in this regard by exploring, for example, changes to our code and materials deposition policies. Excellent author service has been and will continue to be an important priority for *PLOS Biology*, with its unique symbiosis of academic and professional editors that ensure expertise, fairness and efficiency. Providing authors with timely and detailed decisions is our priority, as is easing the publication process with, for example, our format-free submission, scooping protection, transparent review and direct preprint-to-submission policies. We believe that transparency, accountability and approachability are essential in the publication process, and authors should feel free to approach us at any stage. As part of our service to authors, we will strengthen our transfer process to other PLOS journals, to ease author experience and facilitate quick decisions at other relevant titles.

The drive behind everything we do is to serve the life science community and help you advance discovery, not only by publishing excellent work but also by driving and advocating for positive change in publishing. We very much look forward to the future!

